# *cis*-Dichlorido­tetra­kis­(dimethyl sulfoxide-κ*O*)chromium(III) chloride dimethyl sulfoxide monosolvate

**DOI:** 10.1107/S160053681301622X

**Published:** 2013-06-15

**Authors:** Nada A. Al-Najjar, Abdul-Razak H. Al-Sudani, Muthana A. Shanshal, Falih H. Mousa, Benson M. Kariuki

**Affiliations:** a Department of Chemistry, College of Science for Women, Baghdad University, Baghdad, Iraq; b Department of Chemistry, College of Science, Baghdad University, Baghdad, Iraq; c Department of Chemistry, College of Education (Ibn Al-Haitham), Baghdad University, Baghdad, Iraq; d School of Chemistry, Cardiff University, Main Building, Park Place, Cardiff CF10 3AT, Wales

## Abstract

The structure of the title compound, [CrCl_2_(C_2_H_6_OS)_4_]Cl·C_2_H_6_OS, consists of a Cr^III^ ion coordinated by four O atoms of dimethyl sulfoxide (DMSO) ligands and two chloride ions in *cis* positions, forming a distorted CrCl_2_O_4_ octa­hedron. An isolated Cl^−^ counter-anion and a positionally disordered DMSO mol­ecule [occupancy ratio 0.654 (4):0.346 (4)] are also present. In the structure, the complex cations inter­act with the Cl^−^ counter-anions and the DMSO solvent mol­ecules *via* weak C—H⋯Cl and C—H⋯O inter­actions, forming a three-dimensional network.

## Related literature

For details of the synthetic procedure, see: Pedersen (1970 ▶). For background to DMSO as a ligand, see: Boschmann & Wollaston (1982 ▶).
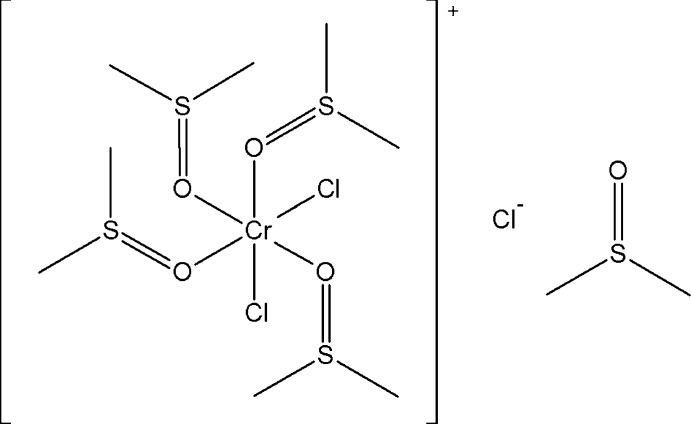


## Experimental

### 

#### Crystal data

[CrCl_2_(C_2_H_6_OS)_4_]Cl·C_2_H_6_OS
*M* *_r_* = 548.99
Triclinic, 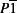

*a* = 9.4521 (2) Å
*b* = 11.0048 (3) Å
*c* = 12.9761 (2) Å
α = 100.501 (2)°
β = 109.007 (1)°
γ = 98.427 (1)°
*V* = 1223.62 (5) Å^3^
*Z* = 2
Mo *K*α radiation
μ = 1.24 mm^−1^
*T* = 150 K
0.20 × 0.20 × 0.20 mm

#### Data collection

Nonius KappaCCD diffractometer
Absorption correction: multi-scan (*DENZO* and *SCALEPACK*; Otwinowski & Minor, 1997 ▶) *T*_min_ = 0.790, *T*_max_ = 0.790
8105 measured reflections
5946 independent reflections
4673 reflections with *I* > 2σ(*I*)
*R*_int_ = 0.028

#### Refinement

*R*[*F* ^2^ > 2σ(*F* ^2^)] = 0.051
*wR*(*F* ^2^) = 0.138
*S* = 1.11
5946 reflections
263 parameters
104 restraints
H-atom parameters constrained
Δρ_max_ = 0.60 e Å^−3^
Δρ_min_ = −0.78 e Å^−3^

### 

Data collection: *COLLECT* (Nonius, 2000 ▶); cell refinement: *SCALEPACK* (Otwinowski & Minor, 1997 ▶); data reduction: *DENZO* (Otwinowski & Minor, 1997 ▶) and *SCALEPACK*; program(s) used to solve structure: *SIR92* (Altomare *et al.*, 1993 ▶); program(s) used to refine structure: *SHELXL97* (Sheldrick, 2008 ▶); molecular graphics: *ORTEP-3 for Windows* (Farrugia, 2012 ▶); software used to prepare material for publication: *WinGX* (Farrugia, 2012 ▶) and *ACD/Chemsketch* (Advanced Chemistry Development, 2008 ▶).

## Supplementary Material

Crystal structure: contains datablock(s) I, global. DOI: 10.1107/S160053681301622X/wm2748sup1.cif

Structure factors: contains datablock(s) I. DOI: 10.1107/S160053681301622X/wm2748Isup2.hkl

Additional supplementary materials:  crystallographic information; 3D view; checkCIF report

## Figures and Tables

**Table 1:** Hydrogen-bond geometry (Å, °)

*D*—H⋯*A*	*D*—H	H⋯*A*	*D*⋯*A*	*D*—H⋯*A*
C1—H1*B*⋯Cl3	0.98	2.78	3.682 (4)	154
C2—H2*C*⋯O5^i^	0.98	2.57	3.529 (14)	165
C3—H3*B*⋯Cl3^ii^	0.98	2.83	3.648 (5)	142
C4—H4*B*⋯Cl3^ii^	0.98	2.79	3.616 (4)	143
C4—H4*C*⋯O5	0.98	2.43	3.377 (13)	162
C8—H8*C*⋯Cl3^iii^	0.98	2.80	3.641 (5)	144

Symmetry codes: (i) 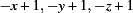; (ii) 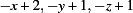; (iii) 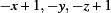.

**Fig. 1.**
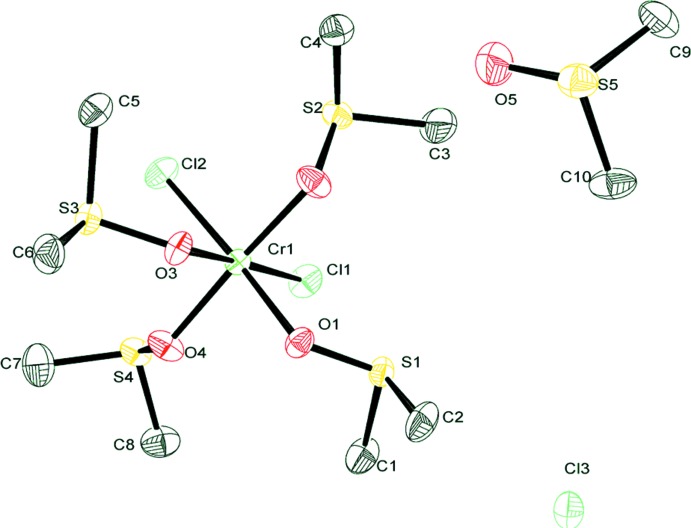
The asymmetric unit of compound (I) with atom labels and displacement ellipsoids at the 50% probability level. Hydrogen atoms and the minor component of the disordered DMSO solvent molecules have been omitted for clarity.

**Fig. 2.**
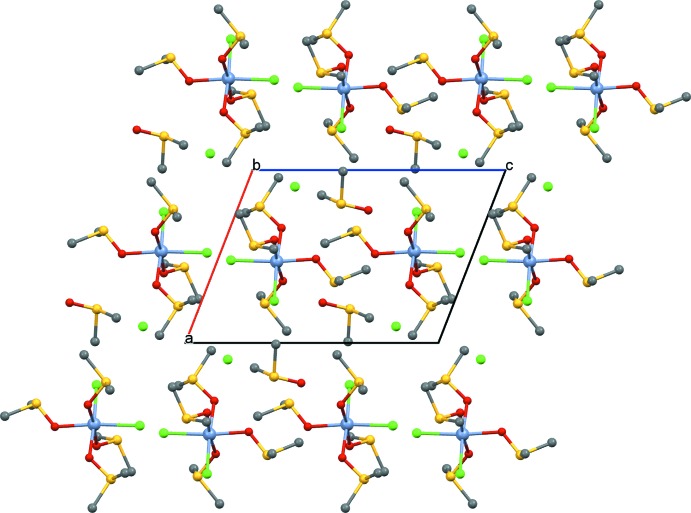
Crystal packing in the structure of (I), with hydrogen atoms and the minor component of the disordered solvent omitted for clarity.

## supplementary crystallographic information

### Comment

*cis*-Dichloridotetrakis(dimethyl sulfoxide-κ*O*)chromium(III) chloride dimethyl sulfoxide monosolvate Metal adducts of aprotic volatile organic solvents have been extensively studied, but the potential of non-volatile aprotic solvent-metal adducts as precursors for useful metal complexes has not been systematically evaluated. The present results are part of a systematic study, including the preparation of anhydrous adducts formed between transition metals salts and non-volatile aprotic solvents (such as DMSO and DMF), their structure, bonding, solubility in common organic solvents, stability in air and the ease at which the coordinating non-volatile solvent molecules can be replaced by other organic molecules. DMSO is an aprotic, highly polar solvent. Its high dielectric constant makes it a good solvent for inorganic as well as organic compounds, and its electronic structure enables it to act as a donor molecule in the formation of coordination complexes with many metal salts. In addition, it can bind to the metal through either the oxygen or sulfur atoms. For examples, see: Boschmann & Wollaston (1982).

The molecular units of the title compound, [CrCl_2_(C_2_H_6_OS)_4_]Cl^.^C_2_H_6_OS, (I), are shown in Fig. 1. The complex cation consists of a chromium(III) ion coordinated by the oxygen atoms of four DMSO molecules with Cr—O distances in the range 1.978 (2)–1.996 (2) Å and two *cis*-positioned chloride ions with Cr—Cl distances of 2.3252 (10) and 2.3302 (9) Å to complete a distorted octahedral geometry. A third and isolated chloride ion balances the charge. An additional uncoordinating DMSO molecule occupies a location displaying disorder with two components with occupancies of 0.654 (4):0.346 (4). In the crystal, the methyl groups of the DMSO ligands interact with the chloride ions and solvent DMSO ligands *via* weak C—H···Cl and C—H···O interactions forming a three-dimensional network (Table 1, Fig. 2).

### Experimental

Complex (I) was prepared by the method described by Pedersen (1970), but on a smaller scale with excess DMSO and other volatile materials removed under dynamic vacuum at 373 K for 5h. The green solid obtained was crystallized by slow diffusion of methanol into a concentrated solution of the complex in DMSO to yield bright green crystals.

### Refinement

The methyl hydrogen atoms have been refined using a riding model with idealized geometry and displacement parameters 1.5 times those of the carbon atoms they are bonded to, and allowed for free rotation. The uncoordinating DMSO molecule is disordered over two sets of sites. Refinement of the disorder (occupancy ratio 0.654 (4):0.346 (4)) has been performed using PART 1, PART 2 and FVAR in *SHELXL* (Sheldrick, 2008). Atoms in close proximity have been refined with identical or similar displacement parameters using SIMU and ISOR instructions in *SHELXL*. The methyl hydrogen atoms for the disordered solvent have been refined using a riding model with staggered idealized geometry and displacement parameters 1.5 times those of the carbon atoms they are bonded to.

### Crystal data

**Table:**

[CrCl_2_(C_2_H_6_OS)_4_]Cl·C_2_H_6_OS	*Z* = 2
*M**_r_* = 548.99	*F*(000) = 570
Triclinic, *P*1	*D*_x_ = 1.490 Mg m^−^^3^
*a* = 9.4521 (2) Å	Mo *K*α radiation, λ = 0.71073 Å
*b* = 11.0048 (3) Å	Cell parameters from 4673 reflections
*c* = 12.9761 (2) Å	θ = 1.7–28.3°
α = 100.501 (2)°	µ = 1.24 mm^−^^1^
β = 109.007 (1)°	*T* = 150 K
γ = 98.427 (1)°	Irregular block, green
*V* = 1223.62 (5) Å^3^	0.20 × 0.20 × 0.20 mm

### Data collection

**Table:**

Nonius KappaCCD diffractometer	5946 independent reflections
Radiation source: fine-focus sealed tube	4673 reflections with *I* > 2σ(*I*)
Graphite monochromator	*R*_int_ = 0.028
ω and phi scans	θ_max_ = 28.3°, θ_min_ = 1.7°
Absorption correction: multi-scan (*DENZO* and *SCALEPACK*; Otwinowski & Minor, 1997)	*h* = −10→12
*T*_min_ = 0.790, *T*_max_ = 0.790	*k* = −14→11
8105 measured reflections	*l* = −17→17

### Refinement

**Table:**

Refinement on *F*^2^	Secondary atom site location: difference Fourier map
Least-squares matrix: full	Hydrogen site location: inferred from neighbouring sites
*R*[*F*^2^ > 2σ(*F*^2^)] = 0.051	H-atom parameters constrained
*wR*(*F*^2^) = 0.138	*w* = 1/[σ^2^(*F*_o_^2^) + (0.0557*P*)^2^ + 2.3503*P*] where *P* = (*F*_o_^2^ + 2*F*_c_^2^)/3
*S* = 1.11	(Δ/σ)_max_ = 0.001
5946 reflections	Δρ_max_ = 0.60 e Å^−^^3^
263 parameters	Δρ_min_ = −0.78 e Å^−^^3^
104 restraints	Extinction correction: *SHELXL*, Fc^*^=kFc[1+0.001xFc^2^λ^3^/sin(2θ)]^-1/4^
Primary atom site location: structure-invariant direct methods	Extinction coefficient: 0.025 (2)

### Special details

**Table:**

Geometry. All s.u.'s (except the s.u. in the dihedral angle between two l.s. planes) are estimated using the full covariance matrix. The cell s.u.'s are taken into account individually in the estimation of s.u.'s in distances, angles and torsion angles; correlations between s.u.'s in cell parameters are only used when they are defined by crystal symmetry. An approximate (isotropic) treatment of cell s.u.'s is used for estimating s.u.'s involving l.s. planes.
Refinement. Refinement of *F*^2^ against ALL reflections. The weighted *R*-factor *wR* and goodness of fit *S* are based on *F*^2^, conventional *R*-factors *R* are based on *F*, with *F* set to zero for negative *F*^2^. The threshold expression of *F*^2^ > 2σ(*F*^2^) is used only for calculating *R*-factors(gt) *etc*. and is not relevant to the choice of reflections for refinement. *R*-factors based on *F*^2^ are statistically about twice as large as those based on *F*, and *R*- factors based on ALL data will be even larger.

### Fractional atomic coordinates and isotropic or equivalent isotropic displacement parameters (Å^2^)

**Table:**

	*x*	*y*	*z*	*U*_iso_*/*U*_eq_	Occ. (<1)
C1	0.6408 (5)	0.1645 (4)	0.5124 (3)	0.0366 (9)	
H1A	0.5359	0.1255	0.5031	0.055*	
H1B	0.7110	0.1660	0.5876	0.055*	
H1C	0.6712	0.1153	0.4555	0.055*	
C2	0.5764 (5)	0.3800 (4)	0.6014 (3)	0.0363 (9)	
H2A	0.5719	0.4688	0.6039	0.054*	
H2B	0.6441	0.3735	0.6747	0.054*	
H2C	0.4731	0.3296	0.5836	0.054*	
C3	0.9311 (5)	0.5941 (5)	0.3820 (4)	0.0614 (15)	
H3A	0.9016	0.6516	0.4345	0.092*	
H3B	1.0167	0.6406	0.3678	0.092*	
H3C	0.9627	0.5244	0.4146	0.092*	
C4	0.7279 (4)	0.6764 (4)	0.2262 (3)	0.0325 (8)	
H4A	0.6327	0.6586	0.1606	0.049*	
H4B	0.8117	0.7244	0.2111	0.049*	
H4C	0.7146	0.7263	0.2918	0.049*	
C5	0.2475 (4)	0.4740 (3)	0.0385 (3)	0.0305 (7)	
H5A	0.2489	0.5476	0.0945	0.046*	
H5B	0.1664	0.4678	−0.0335	0.046*	
H5C	0.3469	0.4836	0.0291	0.046*	
C6	0.0443 (4)	0.3587 (4)	0.1141 (3)	0.0335 (8)	
H6A	0.0164	0.2957	0.1526	0.050*	
H6B	−0.0407	0.3499	0.0434	0.050*	
H6C	0.0655	0.4440	0.1622	0.050*	
C7	0.2549 (5)	−0.0618 (4)	0.0167 (4)	0.0436 (10)	
H7A	0.2169	−0.0017	−0.0270	0.065*	
H7B	0.1832	−0.0881	0.0527	0.065*	
H7C	0.2634	−0.1362	−0.0335	0.065*	
C8	0.4565 (5)	−0.1004 (4)	0.2049 (4)	0.0401 (9)	
H8A	0.5503	−0.0684	0.2719	0.060*	
H8B	0.4620	−0.1809	0.1611	0.060*	
H8C	0.3675	−0.1136	0.2278	0.060*	
Cr1	0.52927 (6)	0.29891 (5)	0.23597 (4)	0.01952 (15)	
O1	0.5147 (3)	0.3118 (2)	0.38722 (19)	0.0249 (5)	
O2	0.6420 (3)	0.4782 (2)	0.2902 (2)	0.0311 (6)	
O3	0.3352 (3)	0.3613 (2)	0.20325 (18)	0.0244 (5)	
O4	0.4037 (3)	0.1239 (2)	0.1927 (2)	0.0307 (6)	
S1	0.64885 (9)	0.32182 (8)	0.49640 (7)	0.02458 (19)	
S2	0.77235 (10)	0.53209 (8)	0.25343 (7)	0.0274 (2)	
S3	0.21164 (9)	0.33420 (8)	0.08474 (7)	0.02458 (19)	
S4	0.43771 (10)	0.01195 (8)	0.12109 (7)	0.0277 (2)	
Cl1	0.75712 (10)	0.22914 (9)	0.28666 (8)	0.0336 (2)	
Cl2	0.52160 (10)	0.28710 (9)	0.05303 (7)	0.0309 (2)	
C9	0.9921 (7)	1.0231 (6)	0.6406 (6)	0.0398 (14)	0.654 (4)
H9A	0.9881	1.0883	0.5980	0.060*	0.654 (4)
H9B	1.0624	0.9710	0.6260	0.060*	0.654 (4)
H9C	1.0286	1.0637	0.7211	0.060*	0.654 (4)
C10	0.8550 (7)	0.8235 (7)	0.6883 (6)	0.0366 (14)	0.654 (4)
H10A	0.7633	0.7602	0.6771	0.055*	0.654 (4)
H10B	0.8980	0.8732	0.7667	0.055*	0.654 (4)
H10C	0.9314	0.7805	0.6712	0.055*	0.654 (4)
O5	0.7674 (13)	0.8501 (11)	0.4809 (10)	0.055 (3)	0.654 (4)
S5	0.80493 (17)	0.92523 (14)	0.59820 (13)	0.0339 (5)	0.654 (4)
C9A	0.875 (3)	0.918 (2)	0.6986 (15)	0.091 (6)	0.346 (4)
H9A1	0.9033	1.0102	0.7138	0.136*	0.346 (4)
H9A2	0.9515	0.8882	0.7538	0.136*	0.346 (4)
H9A3	0.7741	0.8927	0.7042	0.136*	0.346 (4)
C10A	0.812 (2)	0.690 (2)	0.5904 (18)	0.113 (9)	0.346 (4)
H10D	0.7495	0.6273	0.5200	0.170*	0.346 (4)
H10E	0.7535	0.6991	0.6402	0.170*	0.346 (4)
H10F	0.9055	0.6622	0.6272	0.170*	0.346 (4)
O5A	0.723 (3)	0.870 (3)	0.482 (3)	0.065 (6)	0.346 (4)
S5A	0.8659 (4)	0.8479 (4)	0.5591 (3)	0.0520 (11)	0.346 (4)
Cl3	0.90410 (10)	0.28439 (9)	0.80708 (8)	0.0360 (2)	

### Atomic displacement parameters (Å^2^)

**Table:**

	*U*^11^	*U*^22^	*U*^33^	*U*^12^	*U*^13^	*U*^23^
C1	0.052 (2)	0.0291 (18)	0.0280 (18)	0.0178 (17)	0.0096 (17)	0.0064 (14)
C2	0.050 (2)	0.038 (2)	0.0191 (16)	0.0157 (17)	0.0100 (16)	0.0019 (14)
C3	0.034 (2)	0.077 (4)	0.058 (3)	−0.007 (2)	−0.008 (2)	0.040 (3)
C4	0.0353 (19)	0.0313 (18)	0.0329 (18)	0.0094 (15)	0.0128 (16)	0.0105 (15)
C5	0.0328 (18)	0.0331 (19)	0.0272 (17)	0.0133 (15)	0.0089 (15)	0.0101 (14)
C6	0.0213 (17)	0.041 (2)	0.0347 (19)	0.0072 (15)	0.0056 (15)	0.0091 (16)
C7	0.039 (2)	0.042 (2)	0.041 (2)	0.0104 (18)	0.0091 (18)	−0.0039 (18)
C8	0.056 (3)	0.0290 (19)	0.044 (2)	0.0157 (18)	0.025 (2)	0.0116 (17)
Cr1	0.0207 (3)	0.0202 (3)	0.0191 (3)	0.00655 (19)	0.0082 (2)	0.00480 (19)
O1	0.0209 (11)	0.0345 (13)	0.0215 (11)	0.0094 (9)	0.0081 (9)	0.0091 (10)
O2	0.0325 (13)	0.0257 (12)	0.0369 (14)	0.0002 (10)	0.0213 (11)	0.0009 (10)
O3	0.0249 (11)	0.0309 (12)	0.0170 (10)	0.0115 (9)	0.0058 (9)	0.0043 (9)
O4	0.0356 (13)	0.0204 (12)	0.0403 (14)	0.0031 (10)	0.0244 (12)	0.0002 (10)
S1	0.0223 (4)	0.0267 (4)	0.0205 (4)	0.0047 (3)	0.0031 (3)	0.0046 (3)
S2	0.0247 (4)	0.0260 (4)	0.0330 (4)	0.0046 (3)	0.0126 (3)	0.0084 (3)
S3	0.0244 (4)	0.0258 (4)	0.0198 (4)	0.0079 (3)	0.0037 (3)	0.0025 (3)
S4	0.0324 (4)	0.0221 (4)	0.0309 (4)	0.0047 (3)	0.0181 (4)	0.0015 (3)
Cl1	0.0277 (4)	0.0448 (5)	0.0360 (5)	0.0190 (4)	0.0145 (4)	0.0146 (4)
Cl2	0.0394 (5)	0.0377 (5)	0.0229 (4)	0.0171 (4)	0.0162 (3)	0.0092 (3)
C9	0.042 (3)	0.025 (3)	0.048 (4)	−0.003 (2)	0.019 (3)	0.004 (2)
C10	0.029 (3)	0.046 (4)	0.049 (4)	0.014 (3)	0.021 (3)	0.028 (3)
O5	0.065 (7)	0.053 (5)	0.034 (4)	−0.008 (4)	0.014 (5)	0.004 (3)
S5	0.0329 (8)	0.0317 (8)	0.0407 (8)	0.0098 (6)	0.0140 (6)	0.0144 (6)
C9A	0.120 (16)	0.108 (17)	0.052 (10)	0.057 (14)	0.024 (10)	0.029 (11)
C10A	0.079 (14)	0.19 (3)	0.091 (15)	0.059 (16)	0.027 (12)	0.058 (17)
O5A	0.064 (12)	0.068 (9)	0.058 (9)	0.032 (8)	0.015 (8)	0.007 (7)
S5A	0.048 (2)	0.056 (2)	0.048 (2)	0.0241 (16)	0.0129 (17)	0.0019 (15)
Cl3	0.0319 (5)	0.0401 (5)	0.0301 (4)	0.0062 (4)	0.0083 (4)	0.0013 (4)

### Geometric parameters (Å, º)

**Table:**

C1—S1	1.774 (4)	C8—H8B	0.9800
C1—H1A	0.9800	C8—H8C	0.9800
C1—H1B	0.9800	Cr1—O2	1.978 (2)
C1—H1C	0.9800	Cr1—O4	1.983 (2)
C2—S1	1.776 (4)	Cr1—O1	1.993 (2)
C2—H2A	0.9800	Cr1—O3	1.996 (2)
C2—H2B	0.9800	Cr1—Cl1	2.3252 (10)
C2—H2C	0.9800	Cr1—Cl2	2.3302 (9)
C3—S2	1.777 (4)	O1—S1	1.536 (2)
C3—H3A	0.9800	O2—S2	1.539 (3)
C3—H3B	0.9800	O3—S3	1.542 (2)
C3—H3C	0.9800	O4—S4	1.543 (2)
C4—S2	1.769 (4)	C9—S5	1.784 (6)
C4—H4A	0.9800	C9—H9A	0.9800
C4—H4B	0.9800	C9—H9B	0.9800
C4—H4C	0.9800	C9—H9C	0.9800
C5—S3	1.776 (4)	C10—S5	1.766 (6)
C5—H5A	0.9800	C10—H10A	0.9800
C5—H5B	0.9800	C10—H10B	0.9800
C5—H5C	0.9800	C10—H10C	0.9800
C6—S3	1.789 (4)	O5—S5	1.494 (12)
C6—H6A	0.9800	C9A—S5A	1.803 (18)
C6—H6B	0.9800	C9A—H9A1	0.9800
C6—H6C	0.9800	C9A—H9A2	0.9800
C7—S4	1.773 (4)	C9A—H9A3	0.9800
C7—H7A	0.9800	C10A—S5A	1.89 (2)
C7—H7B	0.9800	C10A—H10D	0.9800
C7—H7C	0.9800	C10A—H10E	0.9800
C8—S4	1.780 (4)	C10A—H10F	0.9800
C8—H8A	0.9800	O5A—S5A	1.48 (2)
			
S1—C1—H1A	109.5	O4—Cr1—Cl1	92.15 (8)
S1—C1—H1B	109.5	O1—Cr1—Cl1	93.27 (7)
H1A—C1—H1B	109.5	O3—Cr1—Cl1	176.17 (7)
S1—C1—H1C	109.5	O2—Cr1—Cl2	93.13 (8)
H1A—C1—H1C	109.5	O4—Cr1—Cl2	91.89 (8)
H1B—C1—H1C	109.5	O1—Cr1—Cl2	174.31 (7)
S1—C2—H2A	109.5	O3—Cr1—Cl2	91.44 (7)
S1—C2—H2B	109.5	Cl1—Cr1—Cl2	92.34 (4)
H2A—C2—H2B	109.5	S1—O1—Cr1	125.43 (14)
S1—C2—H2C	109.5	S2—O2—Cr1	123.43 (15)
H2A—C2—H2C	109.5	S3—O3—Cr1	123.90 (13)
H2B—C2—H2C	109.5	S4—O4—Cr1	122.32 (14)
S2—C3—H3A	109.5	O1—S1—C1	105.38 (16)
S2—C3—H3B	109.5	O1—S1—C2	102.39 (16)
H3A—C3—H3B	109.5	C1—S1—C2	98.3 (2)
S2—C3—H3C	109.5	O2—S2—C4	102.63 (17)
H3A—C3—H3C	109.5	O2—S2—C3	103.7 (2)
H3B—C3—H3C	109.5	C4—S2—C3	98.8 (2)
S2—C4—H4A	109.5	O3—S3—C5	104.49 (15)
S2—C4—H4B	109.5	O3—S3—C6	102.73 (15)
H4A—C4—H4B	109.5	C5—S3—C6	97.85 (18)
S2—C4—H4C	109.5	O4—S4—C7	102.81 (18)
H4A—C4—H4C	109.5	O4—S4—C8	103.36 (17)
H4B—C4—H4C	109.5	C7—S4—C8	99.6 (2)
S3—C5—H5A	109.5	S5—C9—H9A	109.5
S3—C5—H5B	109.5	S5—C9—H9B	109.5
H5A—C5—H5B	109.5	H9A—C9—H9B	109.5
S3—C5—H5C	109.5	S5—C9—H9C	109.5
H5A—C5—H5C	109.5	H9A—C9—H9C	109.5
H5B—C5—H5C	109.5	H9B—C9—H9C	109.5
S3—C6—H6A	109.5	S5—C10—H10A	109.5
S3—C6—H6B	109.5	S5—C10—H10B	109.5
H6A—C6—H6B	109.5	H10A—C10—H10B	109.5
S3—C6—H6C	109.5	S5—C10—H10C	109.5
H6A—C6—H6C	109.5	H10A—C10—H10C	109.5
H6B—C6—H6C	109.5	H10B—C10—H10C	109.5
S4—C7—H7A	109.5	O5—S5—C10	107.4 (5)
S4—C7—H7B	109.5	O5—S5—C9	106.6 (5)
H7A—C7—H7B	109.5	C10—S5—C9	97.0 (3)
S4—C7—H7C	109.5	S5A—C9A—H9A1	109.5
H7A—C7—H7C	109.5	S5A—C9A—H9A2	109.5
H7B—C7—H7C	109.5	H9A1—C9A—H9A2	109.5
S4—C8—H8A	109.5	S5A—C9A—H9A3	109.5
S4—C8—H8B	109.5	H9A1—C9A—H9A3	109.5
H8A—C8—H8B	109.5	H9A2—C9A—H9A3	109.5
S4—C8—H8C	109.5	S5A—C10A—H10D	109.5
H8A—C8—H8C	109.5	S5A—C10A—H10E	109.5
H8B—C8—H8C	109.5	H10D—C10A—H10E	109.5
O2—Cr1—O4	173.67 (10)	S5A—C10A—H10F	109.5
O2—Cr1—O1	87.70 (10)	H10D—C10A—H10F	109.5
O4—Cr1—O1	86.93 (10)	H10E—C10A—H10F	109.5
O2—Cr1—O3	87.72 (10)	O5A—S5A—C9A	105.5 (13)
O4—Cr1—O3	88.30 (10)	O5A—S5A—C10A	106.5 (14)
O1—Cr1—O3	82.96 (9)	C9A—S5A—C10A	85.9 (8)
O2—Cr1—Cl1	91.50 (8)		

### Hydrogen-bond geometry (Å, º)

**Table:**

*D*—H···*A*	*D*—H	H···*A*	*D*···*A*	*D*—H···*A*
C1—H1*B*···Cl3	0.98	2.78	3.682 (4)	154
C2—H2*C*···O5^i^	0.98	2.57	3.529 (14)	165
C3—H3*B*···Cl3^ii^	0.98	2.83	3.648 (5)	142
C4—H4*B*···Cl3^ii^	0.98	2.79	3.616 (4)	143
C4—H4*C*···O5	0.98	2.43	3.377 (13)	162
C8—H8*C*···Cl3^iii^	0.98	2.80	3.641 (5)	144

Symmetry codes: (i) −*x*+1, −*y*+1, −*z*+1; (ii) −*x*+2, −*y*+1, −*z*+1; (iii) −*x*+1, −*y*, −*z*+1.
